# Social participation, psychological resilience and depression among widowed older adults in China

**DOI:** 10.1186/s12877-023-04168-7

**Published:** 2023-07-24

**Authors:** Xiaomin Li, Tingshuai Ge, Qing Dong, Quanbao Jiang

**Affiliations:** 1 School of Management, Xi’an Polytechnic University, Xi’an, China; 2 Institute for Population and Development Studies, Xi’an Jiaotong University, Xi’an, China; 3 Xi’an Siyuan University, Xi’an, China

**Keywords:** Social participation, Psychological resilience, Depression, Widowed older adults, China

## Abstract

**Background:**

Depression has become a challenging public health problem, and the loss of a spouse is one of the main causes of depression in older adults. Social participation and psychological resilience are protective factors that reduce depressive symptoms in adults. The purpose of this study was to explore the influences of social participation and psychological resilience on the depression of Chinese widowed older adults.

**Methods:**

We carried out a cross-sectional study on 790 community-dwelling widowed older adults in Shaanxi, China, in 2019. A structured face-to-face interview was used to collect data. We used the ordinary least squares model (OLS), the generalized propensity score matching (GPSM) and the mediation model to test the relationship between social participation, psychological resilience and depression.

**Results:**

We find a U-shaped relationship between social participation and depression, with the increase in social participation scores, the average depression level decreased and then increased. The psychological resilience of the widowed older adults was negatively associated with depression. The higher their psychological resilience, the lower their depression level. Among the four factors of psychological resilience, factor 2 (optimism and positive acceptance of change) and factor 3 (secure relationships and tolerance of negative affect) of psychological resilience were protective factors for depression after adjusting for demographic variables and physical health conditions. Psychological resilience plays a mediating role, as evident in factor 2 and factor 3 mediating the relationship between social participation and depression.

**Conclusion:**

An appropriate level of social participation will relieve the depression of widowed older adults. Social participation can reduce the depression level of widowed older adults by improving their psychological resilience. Community and family could reduce depression by intervening in the social participation of widowed older adults. Active social participation is crucial because it has a protective and resilient impact, which can help people recover from the stress of losing a spouse.

## Introduction

The number of widowed older adults will continue to increase as the aging population does in China. Some older people may readily adapt to life after losing their spouse, while others cannot, and may develop mental health issues such as depression and loneliness.

The loss of a spouse is one of the major causes of depression in older people. Widowed adults are more likely to suffer from depression than married adults, and the prevalence rate of clinically diagnosed depression is around 17%–20% during widowhood [30]. In a survey of older adults in rural China, depression prevalence was reported to be 29.80 percent among the widowed and 13.50 percent among those with spouses [53]. Even if there is a time buffer, some older people are unable to eliminate the impact of the loss of a spouse on depression [44]. Therefore, from the perspective of positive psychology, how to alleviate the mental health of widowed older adults, adapt to widowed life, and achieve successful aging has become an important concern.

Psychological resilience is the concept of positive psychology, which is the ability to adapt and rebound in the face of adversity, trauma, tragedy, threat, or other major stressful events [24, 41]. Most research suggests that resilience is a protective characteristic that decreases mental symptoms in adults [7, 11, 39, 43, 48, 52]. Especially in older adults, in the face of the challenges of bodily functions with aging, illness, and role transformation, the older will be more likely to have negative emotions. A study investigated the association between psychological resilience, stressful life events, and depressive symptoms in older Chinese people, and discovered that resilience plays a vital role in reducing the negative impact of stressful life events on depressive symptoms [31]. According to longitudinal research, about half of those who experienced the loss of a spouse reported minimal to no symptoms of depression at any point during the study, which was related to the psychological resilience of widowed people [6].

Social participation is “a socially oriented sharing of individual resources” [9]. It is a positive factor in relieving depression. Most studies have shown that depression can be prevented and alleviated by actively engaging in social participation among older adults [13, 19, 20, 50]. Social participation provides opportunities to interact with others in society. Older Australians have reported less emotional distress with higher social participation than those with lower social participation because they have received more social support and social contact [49]. For widowed older adults, social participation during widowhood may assist individuals in effectively overcoming the problems of spouse bereavement, regardless of how social participation changes after widowhood [27]. A high level of social participation during widowhood is related to a low level of depression.

However, it is not clear how social participation alleviates depression. According to previous studies, social participation improves the chance of contact with society and enhances the individual’s psychological resilience [5, 48], which is a protective factor for depression. Social participation is a crucial factor in promoting psychological resilience. Volunteering and assisting others have been shown to promote resilience [8, 40]. Wermelinger et al. [48] demonstrated that active people have greater resilience than sedentary people among older people. Social participation provides an opportunity for social and communal interaction. More social and community contact is linked to increased resilience in older adults [5]. Higher levels of social and community engagement are the central protective elements of resilience in later life, according to the Annual Report on Strengthening Personal Resilience in East Sussex [33]. Some resilience-building programs for people aged 65 above focus on developing community connections and participating in meaningful activities [14].

**Fig. 1:**
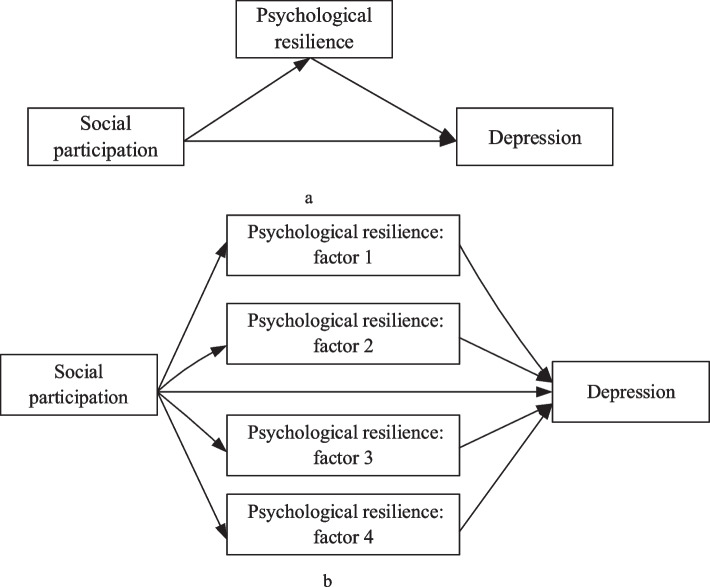
Mediation paths analysis for social participation on the depression. **a** The mediating variable is the total psychological resilience; **b** The mediating variable is the factors of total psychological resilience

Widowed older adults may withdraw from social networks and experience social detachment after losing their spouses. It is critical to encourage active social participation and resilience in widowed older adults in order to reduce isolation and negative feelings. We observed few studies on the relationship between psychological resilience and depression in widowed older adults based on the literature. Although most studies have shown that social participation may alleviate some symptoms of depression, it is unclear how social participation affects depression. We examined the following three relationships in this study:

1. The relationship between social participation and the level of depression among widowed older adults.
2. The relationship between psychological resilience and the level of depression among widowed older adults. Additionally, we examine the relationship between factors of psychological resilience and the level of depression among widowed older adults.
3. The mediating effect of psychological resilience (including the factors of psychological resilience) in the association between social participation and depression. The mediation pathways are shown in Fig. 1.

## Methods

### Sampling

The data was from the “Survey of social adaptation and health among the widowed older adults in Shaanxi Province”, which was conducted in 2019 by the Institute for Population and Social Policy Studies at Xi’an Polytechnic University. Shaanxi Province is in northwest China, and is divided into three regions: central Shaanxi, northern Shaanxi, and southern Shaanxi, all of which have distinct geographical, historical, cultural, and language characteristics [29]. Stratified non-probability sampling was used in the survey. The sample units in the first stage were the district and county in each of the three regions. In the second stage, the sample units were the neighborhood committee and village committees. The sample unit was widowed older adults aged 60 and above in the third stage. According to the proportion of the older population in three regions of Shaanxi Province at 6:3:1, we randomly selected 600 people in central Shaanxi, 300 in southern Shaanxi, and 100 in northern Shaanxi. Because some old people refused to participate in the survey, 900 people were interviewed in total, with 790 questionnaires being valid, including 460 in central Shaanxi, 252 in southern Shaanxi, and 78 in northern Shaanxi. A total of 60 undergraduate interviewers were recruited to conduct the survey during the 2019 summer vacation who had taken training related to social surveys or had some experience with social surveys. The survey was conducted using face-to-face interviews to guarantee its reliability. The data we collected included individual characteristics, health status, social participation, social support, etc.

### Measurement

#### Dependent variables

**Depression** was assessed using the 10-item Center for Epidemiologic Studies Depression Scale (CES-D-10) short form. There are 10 items on the scale. The items relate to the respondent’s feelings and actions over the previous week. There are four options for each question: 0 = never or seldom (1 day); 1 = some or a little (1–2 days); 2 = occasionally or for a long time (3–4 days); and 3 = most or all of the time (5–7 days).

A continuous variable for depression was created by summing up the values of ten items. The total score ranges from 0 to 30, with a higher number indicating a higher level of depression. The study’s Cronbach’s alpha of 0.829 demonstrated its high reliability and validity.

#### Independent variables

##### Psychological resilience

We used the Connor Davidson Resilience Scale (CD-RISC) to assess resilience [12]. It comprises 25 questions, each of which is graded on a 5-point Likert scale (Not true at all,Rarely true; Sometimes true; often true; True nearly all of the time). The sum of the 25-item scores was used to produce a resilience scale, with higher scores indicating stronger levels of resilience. Cronbach’s alpha for this study was 0.9346.

A series of categorical EFAs (Exploratory Factor Analysis) was conducted to determine the factor structure of the CD-RISC scores in the widowed population. The 5- and 3-item solutions were rejected due to numerous cross-loadings and non-meaning factors. The 4-factor structure option was chosen because of its interpretability (Table 1). 57.34% of the variance was explained in this model.

**Table 1:** Rotated factor structure of Connor-Davidson resilience scale

Items	Content	Factor 1	Factor 2	Factor 3	Factor 4
12.	When things look hopeless, I don’t give up	0.789			
11.	Believe I can achieve my goals	0.729			
25.	I take pride in my achievements	0.662			
17.	I think of myself as a strong person	0.660			
16.	Not easily discouraged by failure	0.651			
24.	I work to attain my goals	0.644			
15.	Prefer to take the lead in problem solving	0.609			
18.	Make unpopular or difficult decisions	0.545			
21.	I have a strong sense of purpose	0.491			
23.	I like challenges	0.482			
4.	Can deal with whatever comes		0.732		
6.	See the humorous side of things		0.649		
5.	Past success gives confidence for new challenge		0.623		
7.	Coping with stress strengthens		0.620		
1.	Able to adapt to change		0.581		
8.	Tend to bounce back after illness or hardship		0.563		
13.	Know where to turn for help			0.767	
2.	Close and secure relationships			0.707	
19.	Can handle unpleasant feelings			0.591	
14.	Under pressure, focus and think clearly			0.461	
9.	Things happen for a reason				0.754
22.	In control of your life				0.428
10.	Best effort no matter what				0.399

Coefficients below 0.30 are not displayed in the table (for example: item 3, item 20)

The following factors emerged (see Table1): (1) perseverance and leadership (contains 10 items; The Cronbach’s alpha is 0.9030); (2) optimism and positive acceptance of change (contains 6 items; The Cronbach’s alpha is 0.8443); (3) Secure relationships and distress tolerance (contains 4 items; The Cronbach’s alpha is 0.6703); (4) Control ability (contains 3 items; The Cronbach’s alpha is 0.6241).

The 4-factor CFA provided a good fitting to the data (2/df = 2.610, RMSEA = 0.064, CFI = 0.917, IFI = 0.918, TLI = 0.905). The correlation between resilience and resilience factors was high (0.752–0.933; *P* < 0. 001).

##### Social participation

Social participation was measured with the question, “Have you done these activities in the past 3 months?” Participation in local communities consists of eight components: (1) Interacted with friends; (2) Played Ma-jong, Chinese chess, cards, or went to a neighborhood club; (3) Provided assistance to relatives, friends, or neighbors who do not reside with you and did not pay you for the assistance; (4) Went to sports, social, or other types of club; (5) Participated in a community-based group; (6) Volunteered or donated to charity; (7) Took care of grandkids; (8) Watched TV, read newspapers, and went on the internet. The answers for each option were: 0 = never, 1 = seldom (1 day per week), 2 = some or a little (2–3 days per week), 3 = occasionally or for a significant amount of time (4–5 days per week), and 4 = every day. We summed the values for each option and defined it as “social participation”, a continuous variable with a minimum value of 1 and a maximum of 30, with higher numbers indicating greater participation. Cronbach’s alpha was calculated to be 0.673. Cronbach’s alpha between 0.5 and 0.7 implies moderate dependability, which is also acceptable [4].

#### Control variables

In this paper, we controlled variables such as demographic characteristics, life habits, social support, the number of chronic diseases, and duration of widowhood.

Demographic characteristics include age, gender, education, and personal income, household registration type and work or not. Life habits include, whether the respondent ever smoked or drank. Social support is measured by the number of children and friends. The number of chronic diseases include, hypertension, diabetes or high blood sugar, cancer or malignant tumors, and so on. The summary statistics for all variables used in the analysis are presented in Table 2.

**Table 2:** The descriptive statistics of variables

Variable	Mean value/ Percentage	SD	Definition and measurement
Depression	9.91	5.83	0–29
Social participation	11.56	5.03	1–30
Total CD-RISC	49.83	15.66	5–91
Factor 1: Perseverance and Leadership	19.53	7.87	0–40
Factor 2: Optimism and positive acceptance of change	13.26	4.53	1–24
Factor 3: Secure relationships and distress tolerance	9.29	3.19	0–16
Factor 4: Control ability	7.74	2.34	1–12
Gender	34.1%		0 = male
	65.9%		1 = female
Age group			
60–69 age group	30.52%		0 = 60–69 age group
70–79 age group	46.83%		1 = 70–79 age group
80 age above	22.65%		2 = 80 age above
Education			
Illiterate	46.33%		0 = illiterate
Primary school	33.92%		1 = primary school
Junior high school or above	19.75%		2 = junior high school or above
Personal income (ln+1)	8.92	1.01	5.71–11.29
Work or not	51.38%		0 = without work now
	48.86%		1 = in work now or farming
Household registration type	26.33%		0 = urban resident
	73.67%		1 = rural resident
The number of children	1.43	1.18	0–8
The number of friends	2.47	4.48	0–30
Chronic diseases	1.94	1.05	0–6
Duration of widowhood	12.97	11.06	0–60
Life satisfaction	3.66	0.93	1–5
Smoke	87.47%		0 = no
	12.53%		1 = yes
Drink	83.41%		0 = no
	7.59%		1 = yes

If the first column variable is a continuous variable, the mean value is shown in the second column; if it is a categorical variable, the percentage is shown in the second column

### Statistical analysis

Firstly, we established an OLS (ordinary least squares) regression model to investigate the link between widowed older adults social participation, psychological resilience, and depression.

Secondly, in order to solve the sample selection bias, we use the generalized propensity scores and estimate dose response functions [25]. We build the GPSM (the Generalized Propensity Score Matching) model and examine the effects of different levels of social participation or psychological resilience on depression. Compared with traditional propensity score matching (PSM), the generalized propensity score matching (GPSM) develops a binary treatment into a continuous treatment setting [25, 26].

This approach is suitable for our objective because we are interested in the response—that is, depression namely depression, that is associated with each continuous treatment value, such as the degree of social participation or psychological resiliency. For multivalued treatments, we could discretize the continuously distributed treatment variable and use propensity score methods. The generalized propensity score has the advantage of utilizing all of the information contained in the distribution of treatment duration.

1. We assume the treatment (Ti) is normally distributed when the covariate variables (Xi) are taken into account. We estimate the parameters β0; β1, σ2 by OLS
  $${T}_{i} | {X}_{i}\sim N\left({\beta }_{0}+{\beta }_{1}{\prime}{X}_{i},{\sigma }^{2}\right).$$
2. The GPS is estimated using the following equation:
  $${\widehat{R}}_{i}=\frac{1}{\sqrt{2\pi {\sigma }^{2}}}\mathrm{exp}\left[-\frac{{\left({T}_{i}-{\widehat{\beta }}_{0}-{\widehat{\beta }}_{1}{\prime}{X}_{i}\right)}^{2}}{2{\widehat{\sigma }}^{2}}\right]$$
3. We estimate the expected value of the outcome variable after obtaining the GPS (The generalized propensity score). The quadratic form accounts for a potential non-linear relationship between social participation and depression.
  $$E\left[{Y}_{i}|{T}_{i},{R}_{i}\right]={\alpha }_{0}+{\alpha }_{1}{T}_{i}+{\alpha }_{2}{T}_{i}^{2}+{\alpha }_{3}{R}_{i}+{\alpha }_{4}{R}_{i}^{2}+{\alpha }_{5}{T}_{i}{R}_{i}$$

The average potential outcome at treatment level t is estimated:

$$E\left[\widehat{Y}\left(t\right)\right]=\frac{1}{N}\sum_{i=1}^{N}\left[{\widehat{\alpha }}_{0}+{\widehat{\alpha }}_{1}.t+{\widehat{\alpha }}_{2}.{t}^{2}+{\widehat{\alpha }}_{3}\widehat{r}\left(t,{X}_{i}\right)+{\widehat{\alpha }}_{4}\widehat{r}{\left(t,{X}_{i}\right)}^{2}+{\widehat{\alpha }}_{5}.t.\widehat{r}\left(t,{X}_{i}\right)\right]$$

This is done for every treatment level we are interested obtaining the entire dose-response function.

Lastly, we used Simple Mediation and Multiple Mediation to test whether psychological resilience and four factors acted as mediators between social participation and depression. We used bias-corrected bootstrapping to measure indirect effects [37]. Bootstrapping involves extracting samples from a data set several times (5,000 times in this paper,) and estimating the indirect impact of each resampled data set. The sum of all estimated indirect effects allows for developing of a 95 percent confidence interval for each indirect impact’s effect size. If the confidence interval size includes zero, the effect is considered not significant.

## Results

### Descriptive results

As shown in Table 2, the mean depression level of the widowed older adults was 9.91. The mean level of social participation is 11.56. The mean total score on the CD-RISC in our sample was 49.83, and the average value of the four resilience factors was 19.53, 13.26, 9.29, and 7.74. Among the widowed older adults, 46.83% were 70–79 years old, 46.33% were illiterate, 73.67% lived in rural areas, and 48.86% were still working.

### Regression results

Table 3 presents the regression results. In Model 1, without considering the psychological resilience variable, the higher the level of social participation, the lower the degree of depression, and the social participation variable has a greater impact on the level of depression than other variables.

**Table 3:** OLS estimates for social participation and psychological resilience on depression

Variable	Model 1		Model 2		Model 3	
	β	Beta	β	Beta	β	Beta
Social participation	−0.17*** (0.04)	−0.17	−0.06* (0.04)	−0.06	−0.05 (0.04)	−0.04
Total CD-RISC			−0.16*** (0.01)	−0.43		
Factor 1: Perseverance and leadership					0.00 (0.03)	0.00
Factor 2: Optimism and positive acceptance of change					−0.46*** (0.06)	−0.36
Factor 3: Secure relationships and distress tolerance					−0.26*** (0.07)	−0.14
Factor 4: Control ability					−0.06 (0.09)	−0.02
Gender	0.95** (0.44)	0.07	−0.06 (0.38)	0.01	0.48 (0.39)	0.03
Age group						
70–79 age group	0.17 (0.42)	0.01	−0.37 (0.40)	−0.03	0.00 (0.37)	0.00
80 age above	−0.49 (0.54)	−0.03	−0.82 (0.49)	−0.03	−0.36 (0.47)	−0.02
Education						
Primary school	0.19 (0.43)	0.02	0.22 (0.38)	0.02	0.22 (0.38)	0.02
Junior high school or above	−0.51 (0.54)	−0.03	0.15 (0.49)	0.01	0.17 (0.48)	0.01
Personal income	−0.15 (0.21)	−0.03	0.16 (0.19)	0.03	0.09 (0.19)	0.01
Work or not	−1.31*** (0.41)	−0.11	−0.84*** (0.34)	−0.07	−0.88*** (0.34)	−0.08
Household registration type	0.76 (0.48)	0.06	0.43 (0.43)	0.03	0.34 (0.42)	0.03
The number of children	−0.39*** (0.15)	−0.08	−0.26** (0.14)	−0.05	−0.25* (0.14)	−0.05
The number of friends	0.02 (0.07)	−0.01	0.06 (0.06)	0.03	0.04 (0.06)	0.02
Chronic diseases	0.84*** (0.17)	0.15	0.76*** (0.15)	0.14	0.66*** (0.15)	0.12
Duration of widowhood	0.01 (0.02)	0.02	0.01 ((0.02)	0.02	0.00 (0.01)	0.01
Life satisfaction	−2.19*** (0.20)	−0.35	−1.68*** (0.18)	−0.27	−1.57*** (0.18)	−0.25
Smoke	−0.16 (0.62)	0.01	0.09 (0.56)	0.00	−0.08 (0.55)	−0.00
Drink	1.32* (0.73)	0.06	1.14* (0.66)	0.05	0.97 (0.64)	0.04
_cons	19.69*** (2.23)		21.76*** (2.00)		23.13*** (1.98)	
*AIC*	4779.0		4608.81		4575.92	
*BIC*	4858.5		4692.91		4674.13	
*N*	790		790		790	

^*^p < 0.01; **p < 0.05; ***p < 0.01

Model 2 adds the total score of psychological resilience. The relationship between social participation and depression is still significant. There is a significant correlation between psychological resilience and the degree of depression. The higher the total score of psychological resilience, the lower the degree of depression. After the psychological resilience variable was added to model 2, it had a greater impact on the level of depression than other variables.

Model 3 adds the four factors of psychological resilience. In model 3, social participation was not related to depression, and there was a significant correlation between factor 2, factor 3, and the degree of depression. Factor 2 had the greatest influence on depression, followed by factor 3.

### The dose-response functions estimated using the GPSM

The treatment variables were social participation and psychological resilience. According to the previous analysis of the OLS model, we controlled the personal characteristic variables, social support variables, lifestyle and habits and other variables that affect depression. We believe that these variables affect not only depression but also decisions related to social participation and psychological resilience due to unobserved common variables such as individual ability and environments. Therefore, the GPSM variables are organized using control variables in the OLS model.

### Balancing property

Tables 4 and 5 present the Balancing Property Test for the pretreatment covariates before and after accounting for the estimated GPS (The generalized propensity score). The treatment variable is social participation in Table 4, and the treatment variable is psychological resilience in Table 5.

**Table 4:** Covariates balancing property test before and after matching (Treatment variable is social participation)

Variable	Unadjusted			Adjusted for the GPS		
	[1, 9]	(9, 14]	(14, 30]	[1, 9]	(9, 14]	(14, 30]
	MD (SE)	MD (SE)	MD (SE)	MD (SE)	MD (SE)	MD (SE)
Gender	0.13 (0.29)	0.03 (0.17)	0.23 (0.35)	−0.00 (0.04)	0.01 (0.03)	−0.00 (0.04)
Age group						
70–79 age group	−0.24 (0.28)	0.06 (0.17)	0.37 (0.34)	0.00 (0.04)	0.01 (0.04)	−0.11 (0.04)
80 age above	0.31 (0.29)	0.33 (0.19)	0.38 (0.60)	0.02 (0.02)	0.00 (0.03)	0.09*** (0.04)
Education						
Primary school	−0.35 (0.29)	0.06 (0.18)	−0.18 (0.34)	−0.03 (0.04)	0.01 (0.03)	−0.05 (0.04)
Junior high school or above	−0.29 (0.39)	0.02 (0.21)	0.30 (0.38)	0.03 (0.03)	0.00 (0.03)	−0.04 (0.03)
Personal income	−2.40*** (0.14)	2.98*** (0.09)	8.41*** (0.18)	0.02 (0.07)	−0.01 (0.08)	−0.11 (0.07)
Work or not	−0.39 (0.29)	−0.27 (0.17)	0.01 (0.35)	0.04 (0.03)	−0.09** (0.03)	−0.03 (0.04)
Household registration type	0.89*** (0.34)	0.12 (0.19)	−0.54 (0.36)	−0.05 (0.03)	0.03 (0.03)	0.05 (0.04)
The number of children	4.93 *** (0.15)	10.33 *** (0.11)	16.25*** (0.18)	0.15 (0.09)	−0.21*** (0.08)	0.14 (0.11)
The number of friends	4.81*** (0.16)	9.51*** (0.16)	14.54*** (0.27)	0.63*** (0.22)	−0.22 (0.19)	−0.99 (0.23)
Chronic diseases	4.19*** (0.15)	10.00*** (0.11)	15.82*** (0.18)	−0.11 (0.08)	0.06 (0.08)	0.07 (0.09)
Life satisfaction	2.83*** (0.15)	8.23*** (0.10)	13.71 *** (0.17)	0.04 (0.06)	−0.02 (0.06)	−0.12 (0.08)
Duration of widowhood	−9.33*** (0.75)	−0.26 (0.64)	6.93*** (0.63)	−0.62 (0.77)	1.31 (0.81)	0.59 (0.97)
Smoke	−0.04 (0.39)	−0.17 (0.249)	−0.68 (0.55)	−0.02 (0.02)	−0.01 (0.02)	0.02 (0.03)
Drink	−1.04 * (0.56)	−0.89 *** (0.31)	−0.55 (0.60)	−0.01 (0.02)	−0.01 (0.01)	0.01 (0.02)

Mean Difference (*MD*) (Standard error, *SE*, in parenthesis)

*p < 0.01; **p < 0.05; ***p < 0.01

**Table 5:** Covariates balancing property test before and after matching (Treatment variable is psychological resilience)

Variable	Unadjusted			Adjusted for the GPS		
	[5, 42]	[43, 57]	[58, 91]	[5, 42]	[43, 57]	[58, 91]
	MD (SE)	MD (SE)	MD (SE)	MD (SE)	MD (SE)	MD (SE)
Gender	1.65 (0.94)	0.82 (0.62)	1.47 (0.96)	0.05 (0.03)	−0.09*** (0.03)	0.08*** (0.03)
Age group						
70–79 age group	0.62 (0.88)	0.51 (0.55)	−0.06 (0.96)	0.03 (0.04)	−0.05 (0.03)	0.03 (0.04)
80 age above	0.24 (1.00)	0.14 (0.67)	−1.68 (1.21)	−0.01 (0.03)	0.01 (0.03)	0.00 (0.03)
Education						
Primary school	0.19 (0.97)	−0.21 (0.57)	2.14** (0.99)	0.03 (0.03)	−0.05 (0.03)	0.02 (0.03)
Junior high school or above	−1.84 (1.32)	−1.13 (0.72)	−2.67 ** (1.04)	0.01 (0.03)	0.02 (0.02)	−0.08*** (0.02)
Personal income	24.55 *** (0.43)	41.09*** (0.27)	58.47*** (0.47)	0.11 (0.06)	−0.02 (0.058)	−0.28 (0.06)
Work or not	−3.21*** (0.91)	−0.49 (0.56)	0.44 (0.97)	0.07 (0.04)	−0.10 (0.03)	−0.01 (0.04)
Household registration type	1.26 (1.20)	0.76 (0.64)	2.21*** (0.97)	−0.04 (0.03)	−0.01 (0.02)	0.12*** (0.03)
The number of children	31.82*** (0.44)	48.58*** (0.28)	66.21 ** (0.48)	0.04 (0.09)	−0.01 (0.08)	−0.19** (0.09)
The number of friends	31.24*** (0.44)	47.94*** (0.31)	64.91*** (0.49)	0.02 (0.22)	0.25 (0.19)	−0.74*** (0.21)
Chronic diseases	31.02*** (0.44)	48.11*** (0.28)	65.98*** (0.48)	−0.06 (0.08)	0.04 (0.07)	0.04 (0.08)
Life satisfaction	29.80*** (0.43)	46.26*** (0.27)	63.83*** (0.48)	0.04 (0.05)	−0.14*** (0.06)	−0.11 (0.06)
Duration of widowhood	18.53*** (0.90)	37.56*** (0.71)	56.03***(0.79)	0.02 (0.85)	0.74 (0.81)	0.98 (0.91)
Smoke	−0.46 (1.29)	0.22 (0.89)	−1.25 (1.41)	−0.02 (0.02)	0.02 (0.02)	−0.02 (0.02)
Drink	−2.49 (1.69)	−0.47 (1.12)	1.65 (1.67)	−0.02 (0.02)	0.02 (0.02)	−0.01 (0.02)

Mean Difference (*MD*) (Standard error, *SE*, in parenthesis)

*p < 0.01; **p < 0.05; ***p < 0.01

In Table 4, the balancing assumption ensures a balanced mean of pre-existing characteristics of widowed people at each social participation interval. Among those widowed people, we divide the range of social participation into three treatment intervals with each interval accounting for approximately 33% of the entire range. The 3 groups correspond to low, medium, and high levels of social participation, to allow for sensible outcome comparisons which is in line with the purpose of our study. More specifically, we define the treatment interval as [1, 9], (9, 14], (14, 30]. The pretreatment covariates are usually very different between observations at different social participation levels. Conditional on the estimated GPS, the adjusted means of pretreatment covariates between observations at each treatment level should not be statistically different. The GPS matching process in Table 5 is the same as in Table 4. Based on the distribution of psychological resilience scores, the treatment interval is defined as [5, 42], (42, 57] and (57, 91].

We can observe in Tables 4 and 5 that the differences in the pretreatment covariates are mitigated after controlling for the estimated GPS. According to a standard two-sided t-test, the balancing property is satisfied at a level lower than 0.01.

### Estimation of the dose-response and treatment functions

We can use the GPS to remove bias associated with differences in covariates. we estimate the dose-response function at each specific level of the treatment, by averaging the conditional expectation function over the GPS at that particular level of the treatment.

**Fig. 2:**
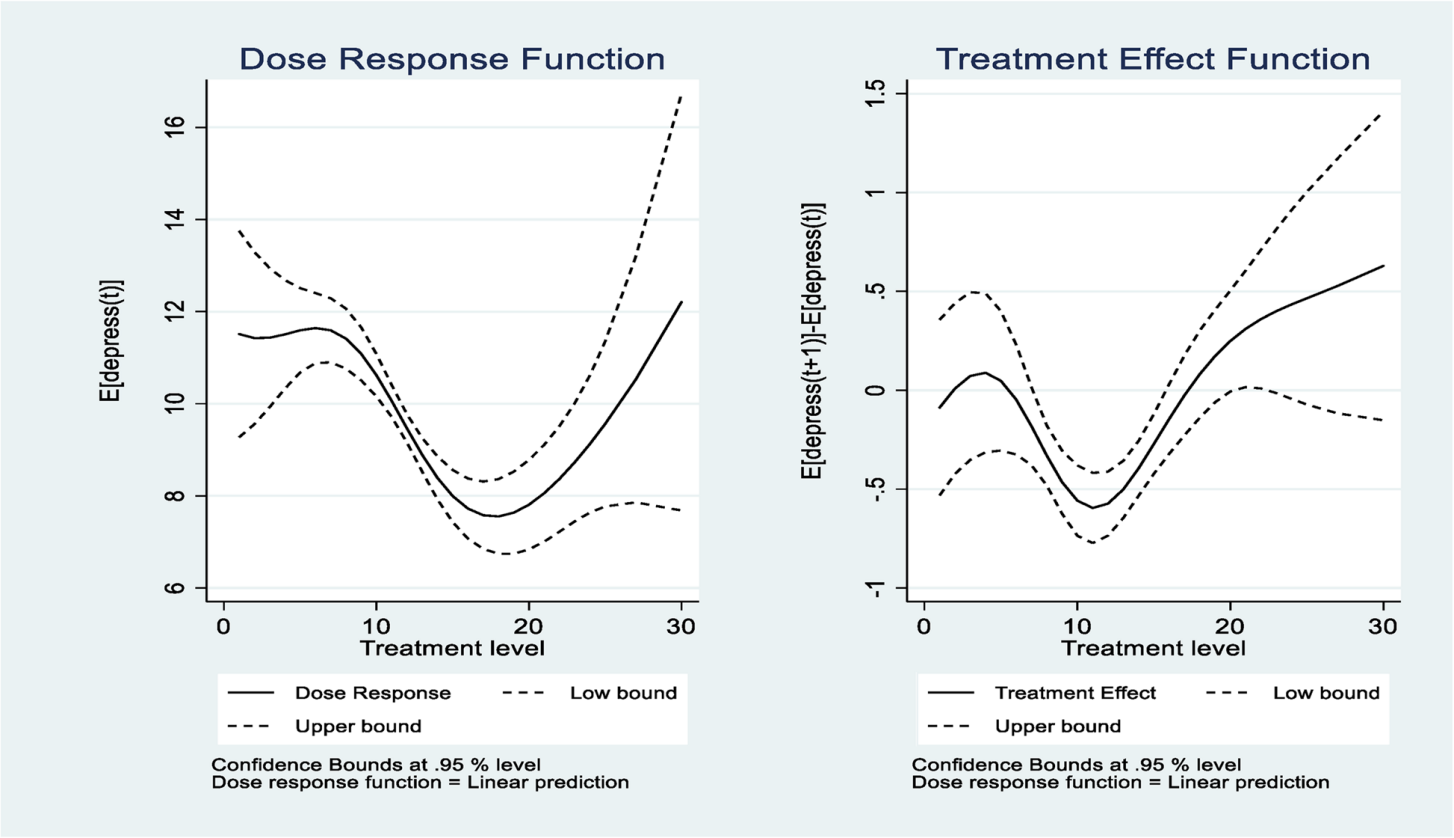
Dose-response function and marginal treatment effect function for depression. (The treatment variable is social participation)

The left panel of Fig. 2 shows the average dose-response function of the social participation score for widowed people and the 95% confidence bands. Moreover, we can see the dose-response function takes on a U shape. With the increase in social participation scores, the average depression level decreased and then increased. When the social participation score was 18, the average depression was the lowest, the social participation score was higher than 18, the average depression began to rise. From the right panel of Fig. 2, it is worth noting that the CI (Confidence interval) for the marginal treatment effect function crosses zero when the social participation score is higher than 18, which means that the marginal treatment effect is insignificant beyond zero point. The right panel of Fig. 2 reveals that marginal effects are larger for high levels of the social participation score.

**Fig. 3:**
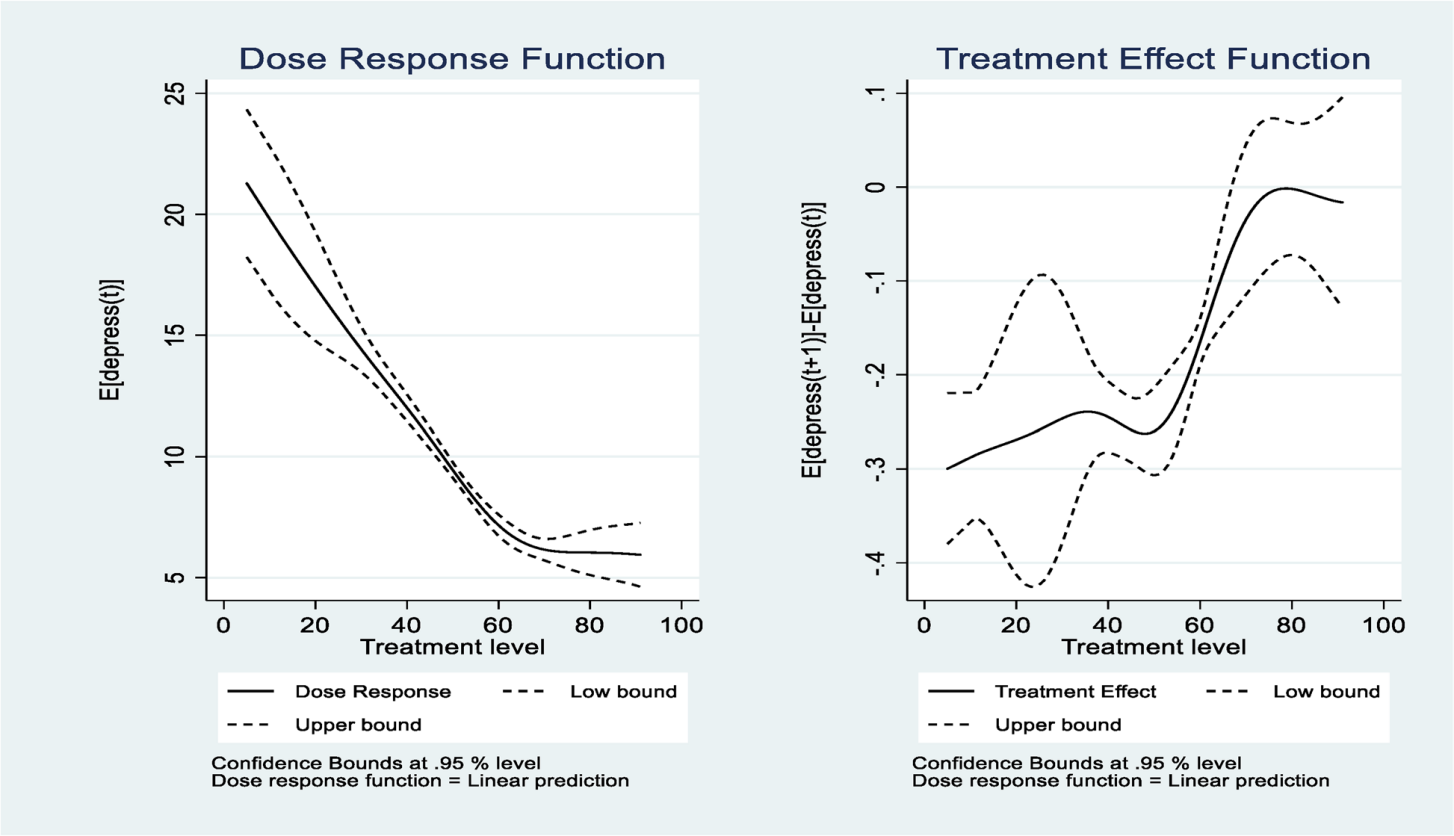
Dose-response function and marginal treatment effect function for depression. (The treatment variable is psychological resilience)

The left panel of Fig. 3 shows the average dose-response function of the psychological resilience score for widowed people and the 95% confidence bands. We can see the average depression is a decreasing function of the psychological resilience score. The relationship between the psychological resilience score and depression is linearly decreasing. The right panel of Fig. 3 reveals that marginal effects are larger for high levels of the psychological resilience score.

### The results of mediation tests

**Fig. 4:**
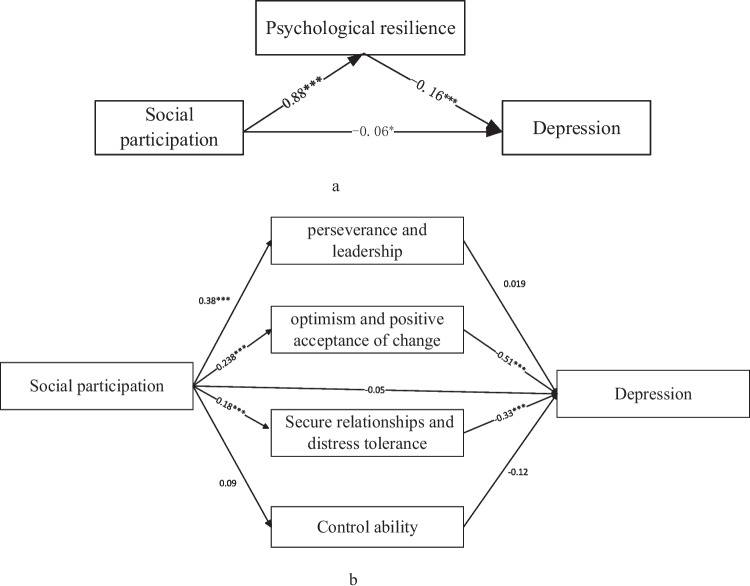
The results of mediation paths analysis for social participation on the depression. **a** The mediating variable is the total psychological resilience; **b** The mediating variable is the factors of total psychological resilience. *p < 0.05; **p < 0.01; ***p < 0.001

Table 6 shows the results of the simple and multiple mediation tests. The simple mediation shows that the indirect effect of psychological resilience was significant (-0.19,-0.10), and the indirect was -0.15. The multiple mediation shows that the total indirect effect for all four mediators assessed simultaneously was significant (-0.24, -0.13); the indirect effect was -0.18. Among the four mediation paths, optimism and positive acceptance of change (-0.17, -0.08), secure relationships, and distress tolerance (-0.09, -0.03) were significant mediation effects. The indirect effects were -0.12 and -0.06, respectively. Higher levels of social participation were associated with lower levels of depression, mediated by factor 2 (optimism and positive acceptance of change) and factor 3 (secure relationships and distress tolerance) of psychological resilience. The point estimates for these specific indirect effects on depression with the psychological resilience factors are detailed in Fig. 4.

**Table 6:** The results of mediation tests

Mediators	Product of coefficients		Bootstrapping Bias-corrected 95% CI	
	Point estimate	Boot SE	Lower	Higher
Total CD-RISC	−0.15	0.02	−0.19	−0.10
Four factors of CD-RISC				
**Facor1:** Perseverance and Leadership	0.01	0.01	−0.02	0.03
**Facor2:** Optimism and positive acceptance of change	−0.12	0.02	−0.17	−0.08
**Facor3:** Secure relationships and distress tolerance	−0.06	0.01	−0.09	−0.03
**Facor4:** Control ability	−0.01	0.01	−0.03	0.01
Total	−0.18	0.03	−0.24	−0.13

## Discussion

We identified that there was a U-shaped relationship between social participation and depression. Several previous studies indicate a linear relationship between social participation and depression, and social participation decreases the level of depression [49, 50]. Our research unveiled that as social participation increases, depression levels initially decline but later increase. Social participation is associated with depression, however, the strength and direction of the association depend on the type of activity [13, 28].

For widowed older adults, social participation provides opportunities for them to integrate into society, strengthen social connections, and reduce social isolation through activities such as volunteer work, cultural activities, leisure activities, sports, and other activities [38, 45]. Leisure activities allow widowed people to cope with their grief while providing an opportunity to experience positive feelings [28]. However, participation in a political or community organization was related to an increase in depressive symptoms. They exert more effort but receive a lower reward, which may result in depressive symptoms [13]. People who watch more television and use computers are more likely to suffer from depression because they are sedentary and lack social support [15, 22]. In China, older people who take care of their grandchildren and take on more housework often experience psychological pressures like anxiety and self-denial because their energy and physical strength are not sufficient to handle the repetitive work and high-intensity care requirements [32]. As a result, moderate, rather than excessive social participation beneficial to health.

We also found that total psychological resilience was negatively associated with depression among widowed people in China. Widowed people with higher scores of total psychological resilience experienced lower levels of depression. People with higher degrees of resilience may be better able to cope with psychological stress because they can remain optimistic despite a life-threatening situation [21]. Resilient people use positive emotions to recover from and find positive meaning in life’s challenges. Positive emotions can expand one’s thinking, assist in the formation of positive relationships, and improve psychological adaptability [46].

Among the four factors of psychological resilience, factor 2 (optimism and positive acceptance of change) and factor 3 (secure relationships and tolerance of negative affect) were significantly negatively correlated with the level of depression. Optimists think those good things will happen instead of bad things, and they look forward to the future with positive emotions rather than negative ones. Optimism and positive acceptance of change were found to be significantly linked to a decreased risk of depression in adults [17],when faced with complex life events, Optimists may have the confidence and courage to face challenges. They can adopt positive acceptance of change in stressful life events [18, 36]. Secure relationships mainly come from the support of family and friends. The recently widowed were less depressed when they had confidence and mutual help from family and friends [16]. After losing a spouse, increased contact with friends and family reduces loneliness, which is thought to be a way of coping with bereavement [47]. Widowed people with more social support from family and friends experience fewer episodes of depression [3]. Low distress tolerance has been linked to greater depressive symptoms [42]. When a person’s ability to tolerate negative emotions is insufficient, they will typically engage in avoidant behaviors [34], which are associated with depression [10, 23].

We found that the association between social participation and the level of depression is mediated by psychological resilience. Active social participation will promote psychological resilience, and the higher the level of psychological resilience, the lower the level of depression. Factors 2 (optimism and positive acceptance of change) and 3 (secure relationships and distress tolerance) of psychological resilience had significant mediation effects among the four mediation pathways. Active social participation affects optimism and positive acceptance of change. For example, volunteering may enhance self-esteem and promote self-worth among volunteers, and it may have an influence on optimism [8, 35]. At the same time,active social participation affects secure relationships and distress tolerance. Such activities as volunteering, caring for grandchildren, and doing community service all contribute to the formation of social networks [33]. Positive social networks are more likely to get social support from family, friends, and neighbors [1]. Secure relationships with family or other trusted adults are positively associated with psychological resilience [2], then high resilience decreases depression. Yoshikawa et al. [54] also found that physical activity strengthens social connections and social support,psychological resilience mediates the association between physical activity and depression,security relationships promote the ability to tolerate negative emotions.

However, we did not find an indirect mediation effect of social participation on depression via factor 1 (perseverance and leadership). It is possible that perseverance and leadership are relatively constant mental states throughout a person’s life, and social participation has little impact on their development. We also found that the mediation path of factor 4 (control ability) is insignificant. Widowhood may undermine one’s feeling of control over events as well as one’s future goals, putting one’s well-being in danger [51].

There are some limitations in the study: Firstly, the data is cross-sectional, and the methods do not resolve reverse causality. Secondly, the retrospective survey of widowed older adults may result in biased information. Thirdly, various factors, such as personality and intergenerational support from their children, can influence the mental health of widowed older adults. However, due to data limitations, these factors are not explored in this study. Nevertheless, our findings could aid in the measurement of future interventions aimed at reducing depression in widowed individuals. Active social participation is essential as it has a protective and resilient impact, aiding in the recovery from the stress of losing a spouse.

## Conclusion

There is a U-shaped relationship between social participation and depression among widowed older adults. Social participation reduces depression in widowed older adults, but too many and frequent participation activities increase depression. As a result, widowed older adults should participate in social activities appropriately.

The higher the level of psychological resilience of widowed older adults, the lower the level of depression. Optimism and positive acceptance of change can help reduce depression. The higher the level of secure relationships and tolerance, the lower the level of depression. It is critical for widowed older adults to build up their psychological resilience.

Social participation can reduce the depression level of widowed older adults by improving their psychological resilience. Participation in social activities improves the optimism and positive acceptance of change in widowed older adults, promotes the level of secure relationships and tolerance in widowed older adults, and then reduces the risk of depression.

## Data Availability

The datasets used and/or analysed during the current study are available from the corresponding author upon reasonable request.

## References

[1] Antonucci T, Akiyama H, Takahashi K (2004). Attachment and close relationships across the life span. Attach Hum Dev.

[2] Bellis MA, Hughes K, Ford K, Hardcastle KA, Sharp CA, Wood S, Davies A (2018). Adverse childhood experiences and sources of childhood resilience: a retrospective study of their combined relationships with child health and educational attendance. BMC Public Health.

[3] Bharathi P, Sridevi G, Kumar KB (2015). Depression among widows and widowers. Int J Sci Res.

[4] Bland JM, Altman DG (1997). Statistics notes: Cronbach’s alpha. BMJ Clin Res.

[5] Blane D, Wiggins RD, Montgomery SM, Hildon Z, Netuveli G (2011). Resilience at older ages: the importance of social relations and implications for policy.

[6] Bonanno GA, Wortman CB, Lehman DR (2002). Resilience to loss and chronic grief: a prospective study from preloss to 18-months postloss. J Pers Soc Psychol.

[7] Bouma E, Riese H, Ormel J, Verhulst FC, Oldehinkel AJ (2009). Adolescents’ cortisol responses to awakening and social stress; effects of gender, menstrual phase and oral contraceptives. The trails study. Psychoneuroendocrinology.

[8] Brown SL, Nesse RM, Vinokur AD, Smith DM (2003). Providing social support may be more beneficial than receiving it. Psychol Sci.

[9] Bukov A, Maas I, Lampert T (2002). Social participation in very old age: cross-sectional and longitudinal findings from BASE. J Gerontol B Psychol Sci Soc Sci.

[10] Campbell-Sills L, Barlow DH. Incorporating emotion regulation into conceptualizations and treatments of anxiety and mood disorders. In: Handbook of emotion regulation. New York: Guilford Press; 2007.

[11] Campbell-Sills L, Cohan SL, Stein MB (2006). Relationship of resilience to personality, coping, and psychiatric symptoms in young adults. Behav Res Ther.

[12] Connor KM, Davidson JRT (2003). Development of a new resilience scale: the Connor-Davidson Resilience Scale (CD-RISC). Depress Anxiety.

[13] Croezen S, Avendano M, Burdorf A, van Lenthe FJ (2015). Social participation and depression in Old Age: a fixed-effects analysis in 10 European countries. Am J Epidemiol.

[14] Davies AR, Grey CNB, Homolova L, Bellis MA (2019). Resilience: understanding the interdependence between individuals and communities.

[15] De Wit L, van Straten A, Lamers F, Cuijpers P, Penninx B (2011). Are sedentary television watching and computer use behaviors associated with anxiety and depressive disorders?. Psychiatry Res.

[16] Dimond M, McCance K, King K, Benoliel JQ, Chang BL (1987). Forced residential relocation. West J Nurs Res.

[17] D’Souza JM, Zvolensky MJ, Smith BH, Gallagher MW. The unique effects of hope, optimism, and self-efficacy on subjective well-being and depression in german adults. J Well-Being Assess. 2021:1–15. 10.1007/s41543-021-00037-5.

[18] Folkman S, Moskowitz JT (2000). Positive affect and the other side of coping. Am Psychol.

[19] Glass TA, De Leon CFM, Bassuk SS, Berkman LF (2006). Social engagement and depressive symptoms in Late Life. J Aging Health.

[20] Gu D, Zhu H, Brown T, Hoenig H, Zeng Y (2015). Tourism experiences and self-rated health among older adults in China. J Aging Health.

[21] Guo J, Liu C, Kong D, Solomon P, Fu M (2018). The relationship between PTSD and suicidality among Wenchuan earthquake survivors: the role of PTG and social support. J Affect Disord.

[22] Hamer M, Stamatakis E, Mishra GD (2010). Television- and screen-based activity and mental well-being in adults. Am J Prev Med.

[23] Hayes SC, Strosahl K, Wilson KG, Bissett RT, Pistorello J, Toarmino D (2004). Measuring experiential avoidance: a preliminary test of a working model. Psychol Rec.

[24] Herrman H, Stewart DE, Diaz-Granados N, Berger EL, Jackson B, Yuen T (2011). What is resilience?. Can J Psychiatry.

[25] Hirano K, Imbens GW. The propensity score with continuous treatments. Wiley Ser Probab Stat. 2005:73–84. 10.1002/0470090456.ch7.

[26] Imbens G (2000). The role of the propensity score in estimating dose-response functions. Biometrika.

[27] Isherwood LM, King DS, Luszcz MA (2012). A longitudinal analysis of social engagement in late-life widowhood. Int J Aging Hum Dev.

[28] Janke MC, Nimrod G, Kleiber DA (2008). Leisure activity and depressive symptoms of widowed and married women in later life. J Leis Res.

[29] Jiang Q, Li Y, Sánchez-Barricarte JJ (2016). Fertility intention, son preference, and second childbirth: survey findings from Shaanxi province of China. Soc Indic Res.

[30] Kristiansen CB, Kjær JN, Hjorth P, Andersen K, Prina AM (2019). The association of time since spousal loss and depression in widowhood: a systematic review and meta-analysis. Soc Psychiatry Psychiatr Epidemiol.

[31] Lim ML, Lim D, Gwee X, Nyunt MSZ, Kumar R, Ng TP (2015). Resilience, stressful life events, and depressive symptomatology among older Chinese adults. Aging Ment Health.

[32] Liu Y, Li T, Guo L, Zhang R, Feng X, Liu K (2017). The mediating role of the sleep quality on the relationship between perceived stress and depression among the elderly in urban communities: a cross-sectional study. Public Health.

[33] Lyons C. Strengthening personal resilience in East Sussex. In: Annual report of the Director of Public Health 2015/16. East Sussex County Council; 2016. https://www.eastsussexjsna.org.uk/resources/annual-public-health-report-2015-16-strengthening-personalresilience-in-east-sussex/.

[34] McHugh RK, Otto MW (2011). Domain-general and domain-specific strategies for the assessment of distress intolerance. Psychol Addict Behav.

[35] Mellor D, Hayashi Y, Firth L, Stokes M, Chambers S, Cummins R (2008). Volunteering and well-Being: do self-esteem, optimism, and perceived control mediate the relationship?. J Soc Serv Res.

[36] Nes LS, Segerstrom SC (2006). Dispositional optimism and coping: a meta-analytic review. Pers Soc Psychol Rev.

[37] Preacher KJ, Hayes AF (2008). Asymptotic and resampling strategies for assessing and comparing indirect effects in multiple mediator models. Behav Res Methods.

[38] Shvedko A, Whittaker AC, Thompson JL, Greig CA (2018). Physical activity interventions for treatment of social isolation, loneliness or low social support in older adults: a systematic review and meta-analysis of randomised controlled trials. Psychol Sport Exerc.

[39] Smith BW, Dalen J, Wiggins K, Tooley E, Bernard J (2008). The brief resilience scale: assessing the ability to bounce back. Int J Behav Med.

[40] Southwick SM, Pietrzak RH, Tsai J, Krystal JH, Charney D (2014). Resilience: an update. PTSD Res Q.

[41] Southwick SM, Charney DS (2012). Resilience: the science of mastering life’s greatest challenges.

[42] Starr LR, Davila J (2012). Temporal patterns of anxious and depressed mood in generalized anxiety disorder: a daily diary study. Behav Res Ther.

[43] Taheri-Kharameh Z, Hazavehei MM (2017). Positive religious coping as a predictor for improvement of mental health among university students. BMJ Open.

[44] The Canadian Study of Health and Aging Working Group (2002). Patterns and health effects of caring for people with dementia: the impact of changing cognitive and residential status1. Gerontologist.

[45] Toepoel V (2012). Ageing, leisure, and social connectedness: how could leisure help reduce social isolation of older people?. Soc Indic Res.

[46] Tugade MM, Fredrickson BL, Feldman Barrett L (2004). Psychological resilience and positive emotional granularity: examining the benefits of positive emotions on coping and health. J Pers.

[47] Utz RL, Carr D, Nesse R, Wortman CB (2002). The effect of widowhood on older adults’ social participation. Gerontologist.

[48] Wermelinger Ávila MP, Corrêa JC, Lucchetti ALG, Lucchetti G (2018). The role of physical activity in the association between resilience and mental health in older adults. J Aging Phys Act.

[49] Windsor TD, Hunter ML, Browne-Yung K. Ageing well: building resilience in individuals and communities. Flinders Centre for Ageing Studies, Office for the Ageing; 2015. https://www.sahealth.sa.gov.au/wps/wcm/connect/public+content/sa+health+internet/resources/ageing+well+-+building+resilience+in+individuals+and+communities.

[50] Won S, Kim H (2020). Social participation, health-related behaviour, and depression of older adults living alone in Korea. Asian Soc Work Policy Rev.

[51] Wortman CB, Silver RC, Baltes PB, Baltes MM (1989). Successful mastery of bereavement and widowhood: a life course perspective. successful aging: perspectives from the behavioral sciences.

[52] Wrenn GL, Wingo AP, Moore R, Pelletier T, Gutman AR, Bradley B, Ressler KJ (2011). The effect of resilience on posttraumatic stress disorder in trauma-exposed inner-city primary care patients. J Natl Med Assoc.

[53] Wu XL, Lei LX, Zhou FH, Li CX (2014). Investigation on depression and its influencing factors of widowed older adults in rural areas. Nurs J Chin People’s Lib Army.

[54] Yoshikawa E, Nishi D, Matsuoka YJ (2016). Association between regular physical exercise and depressive symptoms mediated through social support and resilience in Japanese company workers: a cross-sectional study. BMC Public Health.

